# The Intraocular Serous Membrane and the Nature of Glaucoma

**Published:** 1871-12

**Authors:** A. Sichel


BUFFALO

### Medical and Surgical Journal.

VOL. XI. DECEMBER, 1871. No. 5.

### Original Communications.

:o:

ART. I.—*The Intraocular Serous Membrane and the Nature of Glaucoma. By Dr. A. Sichel, Jr.*

Translated from the *Annales d'Oculistique*, BY F. W. ABBOTT, M. D., Ophthalmologist to the Buffalo Free Dispensary.

There are unquestionably, few subjects in the domain of Ophthalmology which have given rise to so many writings of a contradictory nature as Glaucoma. He would doubtless be accused of temerity, who should attempt to raise a corner of the veil with which this question has, for a long time, been enveloped, since this veil has been shown to be almost impenetrable to the most illustrious masters, as Donders, Arlt, and above all Von Graefe. Nevertheless, a recent discovery, and one which appears to me to be of great importance for the theory of Glaucoma, has induced me to write the following pages, which, if they do not definitely decide the question, will at least make the readers of the *Annales d'Oculistcque,* acquainted with an interesting and conscientious article, which from the special nature of the publication in which it appeared, is, perhaps, not at the disposition of all.

But, before entering into the heart of our subject, permit us, without wishing to give here the history of this question, so well known to all, especially since the excellent thesis of Dr. Hauffmanns, to recall the principal phases which the study of glaucoma has undergone; an examination which perhaps will not be devoid of interest.

From all time the obscurity of the developement and the insidious character of this disease, the inefficiency of medical treatment, and finally the absence of lesions clearly appreciable and determinable by "macroscopic" examination, which alone the ancient masters possessed, have successively excited to a high degree the interest of its observers; but we are compelled to see, that, in spite of the most praise-worthy efforts all have encountered the same obstacles, and no one of them has succeeded in furnishing a clear and plausible explanation of the phenomena observed during life. Although the methods of examination during life, as well as after death, were suddenly perfected in an unhoped for manner, there persisted none the less a great obscurity around this subject; and in spite of the most ingenious hypotheses, or the most senseless theories, and perhaps even on account of these latter, a clear and precise interpretation of the phenomena, based upon palpable facts, remained for a long time in the condition of an ardently coveted desideratum.

The reader will excuse us from recuring to the most remote epoch of the knowledge of glaucoma. It appears in truth to have been known from all antiquity although it appears to have been often confounded with certain forms of cataract. It is sufficient for us, for the end which we propose to attain, to recur to the commencement of this century, the epoch when Ophthalmology commenced its truly scientific era.

Beer considered glaucoma, as a consequence of an arthritic vice, and it is not difficult to recognize in that which he describes under the name of arthritic ophthalmia, that which we to-day call acute glaucoma. Moreover the description which he gives of glaucoma in classing it among arthritic diseases, proves clearly the sagacity of his observant spirit, but teaches us nothing as to the nature of the affection.

To Weller is due the first categorical description of the disease which occupies us. He is the first one who speaks of the vision of colored circles around lights, and of the hardness of the globe. But we see, nevertheless that the pathogeny leaves much to be desired, for he adheres to the opinion of Beer, and believes that he is able to affirm that the true seat of the disease is partly in a trouble of the vitreous humour, which according to his dissections, was of a light greenish brown, but above all in the retina whose color was altered and which was for him the essential seat of the disease. He rests, to confirm his dictum, upon the opinion of Wenzel, whose words he cites: "That which is called glaucoma," says Wenzel, "appears to me to be a true affection of the optic nerve, the alteration of which communicates itself to the retina which is the expansion of it."

As to its pathogeny the first clear idea which we encounter is in the work of Mackenzie, who calls particular attention to the hardness of the globe, which he ascribes to a change of consistence of the media of the eye, and which appears to him to be due to an *accumulation of liquid* in the interior of the globe.

A little after, appeared the celebrated work of my father, in which, taking numerous anatomical researches as a basis, he considers glaucoma as the result of an arthritic choroiditis. This opinion very soon made many partisans, notably Arlt and Schroeder Vander Kolk, and later still Lawrence, who consider the disease as a consequence of alterations of the choroid and consecutively of the retina.

From this time many years elapse without the appearance of notable works upon this subject. Nevertheless Stellwag von Carion remarks that besides the choroiditis there exists in glaucoma other alterations, and that the eye may present troubles more or less marked for a long time before it is possible to recognize the presence of a choroiditis. He even goes so far as to state that far from being always the cause of the trouble, the choroiditis is often only the consequence of it.

With him terminates the ante-ophtholmoscopic period. Up to this point all the authors which we have just cited have based their opinion upon the phenomena observed by the naked eye during life, or upon the result of dissections and anatomical observations made without the assistance of the microscope.

Ed. de Jaeger is at the head of the second period. To him is due the first ophthalmoscopic design of an arthritic (glaucomatous) amaurosis, but the changes which the optic papilla has undergone are ascribed by him to a swollen condition of the nervous disc; and we find no indication of the pathogeny of the disease.

It was then that the first work of Von Graefe appeared, in which he both describes the change of form of the optic disc, its *convex* aspect and pointed out for the first time another extremely important sign, viz:] the spontaneous pulsation of the central artery. He observed upon this subject that he had noticed this phenomena in no other affection of the fundus of the eye, and *that he considered it of extreme importance for interpreting the nature of the malady.*

A little after he published a second article, in which he said that the optic disc is not *swollen*, but rather *excavated,* that is to say in place of being *convex* it is *concave.* These two phenomena, the excavation of the disc, and the spontaneous pulsation of the central artery appear to him calculated to lead us to think that the disease is caused by an *exaggeration of the intraocular pressure,* and to the support of this hypothesis he cites some slight ameliorations obtained by the *repeated evacuation of the aqueous humor,* (paracenteses).

Very soon after appeared his third and immortal work upon the cure of glaucoma by iridectomy, a work in which he still seeks to show that the real cause of the trouble is the exaggeration of the intraocular tension, which he does not hesitate to attribute to a *serous hypersecretion* due to an *inflammatory alteration of the uveal tract.*

Some time after appeared the very interesting thesis of Hauffmann's, in which are given the ideas of Prof. Donders, who attributes the development of glaucoma to the exaggeration of the intraocular tension, produced by a serous hypersecretion, which the celebrated Professor of Utrecht attributes to a *neurosis of the secretary ciliary nerves.*

The doctrine of the augmentation of the intraocular pressure, being advanced by such authors, naturally had great success. It was almost universally accepted by all ophthalmologists, with this difference, however, that some hold to the opinion of Von Graefe, others to that of Donders. Nevertheless these opinions, which referred the cause of glaucoma to an intraocular hypersecretion of liquid, were only hypotheses when some extremely important facts became known.

In the month of January, 1867, during my last sojourn at Berlin, there came to Von Graefe an officer of the Russian army suffering from the prodromata of glaucoma, noticably characterized by the presence in the right eye of vision of colored circles around lights. Warned by the professor of the gravity of the affection from which he was suffering, this officer submitted himself the very next day to an iridectomy. The evening after this operation, upon leaving the clinic to return home, Von Graefe and myself entered a carriage. The weather was very cold, 12° Reaumur, and the professor, whose respiratory organs were, alas, very susceptible, had caused the carriage windows to be carefully closed. These were very soon covered with a thick mist. All at once in looking mechanically through one of the windows at a lighted street lamp, Von Graefe observed that the presence of the mist on this glass caused, around the flame of the gas, the presence of a veritable halo, presenting all the colors of the spectrum, the violet occupying the parts nearest the flame, and the red the peripheral parts. This observation was a veritable illumination to him. He attracted my attention to the phenomenon, we confirmed it by several repetitions, and he told me that he thought that the colored circles which glaucomartous patients see might, very likely, be of the same nature He immediately communicated to me his intention, so soon as the condition of the patient would permit, to make him observe the phenomenon. Some days later, when the patient was fully convelascent and his condition permitted him to brave the rigors of the temperature, Von Graefe, under a trivial pretext, asked him to ride with us. All at once and without his having seen it before, Von Graefe called the attention of the officer to one of the gas burners which we met on our way, immediately the patient cried out: "*Good heavens, Doctor, I am attacked again with my disease. I see again the same rain-bows, not only with the eye operated on, but also with the other.*" Von Graefe and myself were immediately seized with a hilarity, which the poor patient couldn't comprehend, but of which he quickly knew the cause when, after having wiped the window, Von Grafe made him notice that the alarming phenomenon immediately disappeared, and reappeared on the contrary when the patient looked through the other window still covered with mist. This experiment clearly proved the presence, in the eye of the glaucomatous, of a product, analagous to the vapor of water, which disappears after an iridectomy.

I know not how it happened that Von Graefe never published this very interesting observation, which, nevertheless, afforded so signal a confirmation to the hypothesis uttered by himself.

Affairs were thus, when there appeared at the commencement of the first number of the sixth volume of the *Archiv fur Mikroskopische Anatomie* of Max Schultze (Bonn, 1870, Max Cohen & Son,) a very interesting article from G. Shwalbe to which we have already made allusion at the commencement of this work.

In reading this article it appeared to me that it ought to bring about a veritable revolution in the study and knowledge of the question which occupies us, and I ask the permission of the readers of the *Annals* to give them a succinct analysis of the part which especially touches our subject. In commencing, the author announces that in the present work he will only speak of the lymphatic vessels of the posterior segment of the eye, waiting until another number to speak of those of the anterior. The ciliary body forms, according to him, the limit between these two segments. To the posterior segment pertain the lymphatic vessels of the retina, the perichoroid space and finally of the tract situated between the internal and external sheath of the optic nerve, which, without communicating with the two proceeding, inosculate in the arachnoid space.

As to the lympathics of the anterior segment, without giving details of their position, the author merely shows that they form a system completely separate from the preceding, and reserves the description of them in detail for his next work.

After this short preamble the author enters upon the exposition of his subject by the study of the supra choroid tissue If we try to separate the vascular membrane of the eye from sclerotic, we perceive at once that these two membranes are intimately united at only two points; about the entrance of the optic nerve and in the neighborhood of the border of the cornea. In all the rest of their extent, these two membranes separate easily, not taking account, be it understood, of the vascular and nervous branchlets which it is necessary to cut. These membranes once separated, we notice that their surfaces, which are in opposition, *have the shine and smoothness of serous membranes.* Moreover, in man and in certain animals we see that there exists sometimes on the internal face of the choroid, after their separation, a floculent tissue, very rich in pigment which united these two membranes the one to the other. This is the tissue which is generally designated now under the name of "supra choroid membrane," and which is considered as a separate layer from the choroid, properly so called. This membrane is, in fact, very sharply distinguished from the choroid by its peculiar tissue, as well as by the appearance of its pigmentary cells, and also by the absence of all blood vessels. As for the so-called *lamina fusca* of the choroid, it is only the portion of the supra choroid membrane which remains adherent to the sclerotic after the separation.

This shining and polished appearance of the internal face of the sclerotic and the external face of the choroid did not escape the ancient anatomists, and Arnold expressed the opinion that the space comprised between the sclerotic and choroid was a true *lymphatic sac*, and that consequently there existed a veritable *intraocular arachnoid membrane.* We will see farther on the justness of Arnold's opinion. Nevertheless, he failed to show that the intimate texture of this intraocular arachnoid coincided with that of the serous membranes. But this proof was impossible by the methods of examination then in use. It was not astonishing, then, that Bruche himself should deny the existence of this intraocular serous membrane in spite of his knowledge of the microscope, because at this time even they had not at their command the present methods examination. Since then the opinion of Arnold has fallen into oblivion.

But by the aid of histological methods in use to-day, it is not difficult to prove that the walls of the space comprised between the sclerotic and the choroid are very analogous to serous membranes. They show, however, the greatest analogy to lymphatic sacs. To prove the justness of this comparison it suffices to show this cavity is lined with a continuous endothelium.*

It is by using the method of preparation in nitrate of silver, invented by Professor Von Recklinghausen, that the author has been able to make apparent the endothelium which lines the walls of the perichoroid sac. For this he made his first preparations of the eyes of white rabbits, whose lack of pigment renders the silver images much more clear and consequently more apparent. Soon after he

*A name given by His to that variety of epithelium which lines serous cavities.

repeated the same preparations in the eyes of other animals, and finally in the human eye. It is easy to isolate the endothelium of the supra choroid membrane itself. We do this the most easily with pieces which have remained for some time in the solution of Muller. After placing them for some minutes in a solution of $\frac{1}{2}$ to $\frac{3}{4}$ per cent. of nitrate of silver, we obtain preparations which show a network of straight lines inosculating in such a manner as to circumscribe polygonal meshes. Some nuclei with a bi-convex surface, and ellipsoidal contour are distributed here and there in this network. Some of these nuclei are even provided with nucleoli. These nuclei and meshes are sometimes identical with those of the endotheliums of the lymphatic sacs of the frog, sometimes with those which are found on the internal face of the lymphatic vessels of the intestinal canal.

The supra choroid membrane, which does not exist during the embryonic period, is scarcely apparent in the new born or in the first periods of life. It becomes more and more apparent with age, which the author attributes to the repeated movements which the choroid makes to obey the wants of the accommodation for near vision. The repeated tractions have a notable influence upon the ulterior developement of the membrane, and ought to bring about this result, that with the progressive development, the choroid would glide more and more easily upon the sclerotic, and that the free spaces existing between the sclerotic and the choroid should increase.

When we have succeeded in isolating this endothelium,and it does not form folds, it is sometimes difficult to perceieve it, it is only by the presence of nuclei that the attention is found to be drawn to it. These nuclei are also sometimes very difficult to recognize, but coloring them with carmine or iodine may yield precious results. In the points where folds are found, we easily perceive that the membrane itself has a scarcely perceptible thickness. It is only in the vicinity of the nuclei that it has more, thence it gradually diminishes. Hence the whole membrane is found to be composed of thicker and thinner portions. The thickest are always found in the vicinity of the nuclei and the thinnest in the middle of the space between two nuclei. In general the nuclei are widely scattered. Nevertheless several ofthem are often found collected into a group as is observed in other serous sacs as the peritoneum. From a chemical point of view this endothelium is very resisting against acetic acid and the solutions of potash. The nuclei become scarcely more visible from acetic acid, but they swell noticably under the influence of the solutions of potash. These are exactly the same reactions as those which, according to Eberth, the endotheliums of the lymphatic sacs of the frog present.

"From all this, it would be necessary to consider these fine vitreous membranes as an endothelium whose cells are intimately united by their borders, and the protoplasm of which is transformed during the course of their development into elastic tissue. Now that we know of the endothelium of the supra choroid membrane, the intimate texture of the elastic lamellae becomes very clear to us. Each one of them is covered upon its free surface by an endothelium and they enclose between their surfaces a network of elastic fibres and of pigment cells."

In reading this part of this beautiful work, so clear and limpid in statement, and of which only a very feeble analysis has been seen here, numerous reflections came to my mind.

In truth, said I, from the instant in which it is demonstrated that there exists in the eye a serous membrane, it is not astonishing that, as is the case with the other membranes of the same order, there may, in certain conditions, be produced a hypersecretion of liquid, as it happens with the peurae, the peritoneum and the synovial membranes of the articulations.

Finally all the phenomena with which the glaucomatous are tormented, whatever they may be, are consequently explained in a clear and precise manner.

What a brilliant consecration did the theory of Von Graefe receive from this! Was it not a new proof of the sagacity and rectitude of his judgment? Did not his genius anticipate the microscope? Of all the pathogenic phenomena nothing even to the very seat of the secretion escaped him, and he was entirely correct in classing glaucoma among serous choroidites. What matters it, in truth, from the clinical point of view, whether the secretion proceeds from such an such a point of the choroid, if it remains true that this membrane furnishes it, and that its accumulation, in the interior of the globe, provokes the exaggeration of the intraocular pressure?

But since, in a question of so great importance, it is necessary to accept no new statement without submitting it to a rigorous examination and to a careful confronting with the clinical facts, it will be necessary, first of all, to see if all the symptoms of the different forms of glaucoma, as well as the diverse premonitory signs of the disease, find their explanation in the discovery of Schwalbe.

Finally another question of extreme importance, that of the mode of action of an iridectomy, may it not be explained in a satisfactory manner?

I commenced reflecting on these different questions. But at the outset, an important premonitory sign, the rapidly progressing presbyopia, which appeared to me incompatible with this theory, delayed my conviction. Nevertheless I finally found the key of the phenomenon, and immediately as a result of this, for myself, a conviction so firm and complete, that I now have only one desire,to make my confreres partake of this conviction, to render a just testimony to my illustrious and regretted master, and, finally, to put a stop, if possible, to the systematic opposition which is made to his doctrine by envious competitors or jealous rivals.

This is that which I am now about to attempt.

PREMONITORY PERIOD OF GLAUCOMA.

1. The first and most important prodroma, the presence of colored circles around artificial lights, appeared to us to be explained conclusively by the history which we have narrated of the Russian officer whom Von Graefe took into his carriage. It is evident that it is to the serous hypersecretion that we must attribute it, and the intraocular liquid fulfills for the glaucomatous the same role which the moisture collected on the glass fulfills for us.

2. The sudden and rapidly increasing *presbyopia*, another important prodroma appears at first, as I have said before, not to accord with this theory. In truth the choroid, *pushed back* in every part by the liquor, ought to be abnormally stretched, and consequently we should rather find *myopia*, from the increase of the curvature of the posterior face of the crystaline lens But let us remember that it is generally at night during sleep, when the *accommodation is relaxed*, that the prodromata commence. It is usually upon their awakening that patients perceive that changes have occurred in their vision. If, then, the choroid is found at this time to be compressed or crowded back, it is not at all astonishing that the accommodation should be partially paralyzed. Moreover we must not forget that muscular fibres have been found in the very tissue of the choroid, whose contractility is certainly trammeled by the compression. There are also some fibres of the muscle of Bruche. Finally the innervation of this muscle must be much obstructed by the direct compression of the ciliary nerves, whether it proceeds from the direct action of the liquid, or, on the contrary, they are strangled in the sclerotic as they pass through it to penetrate the globe. We see, then, that even here the explanation is perfectly satisfactory. The proofs are even, so to speak, superabundant, and we do not see that anything else than the receding of the vision could be produced in this case.

3. *The diminution of the area of the field of vision* is explained by the compression of the retina, whose *ora serrata*, its most delicate and thinnest part, must manifest the first effects.

4. *The spontaneous pulsations of the central artery* were explained by Von Graefe* in a remarkable manner which becomes still more authorative by the verification of his theory, the proof of the possibility of hypersecretion. It is useless then to dwell longer on it here, and I am contented with referring to the memoir of Von Graefe.

5. Finally, the slight and transitory troubles of the vision are sufficiently explained by a slight effusion of liquid, which, at first, only causes phenomena analagous to those which we produce in our own eyes by slightly pressing them circularly with several fingers or with a ring.

Let us pass now to the examination of glaucoma itself.

1. *Acute glaucoma* at the start would be nothing else than the periodical effusion followed by the complete absorption, more or less rapid, of a variable quantity of serous liquid which, occasioning at first, a traction of the ciliary nerves, would induce the neuralgic pains in the whole of the fifth pair, and principally in the ophthalmic branch of Willis. To this traction would very soon succeed compression of the ciliary nerves, having for consequences anesthesia of the cornea and paresis of the iris. With the maximum

*Archiv f. Ophth., vol. 1, No. 2, p. 299 et seq.

of the effusion would coincide the abolition of the sight, whose reestablishment is owing to the reabsorption of the liquid.

2. *Chronic inflammatory glaucoma* would be caused by the sudden effusion of a considerable quantity of liquid, not followed by its complete reabsorption, and characterized on the contrary by successive effusions, occasioning alternations of clearing up and obscuring of the sight. Here is immediate compression more or less energetic of the internal membranes, particularly of the retina, enduring for a variable time, and at greater or smaller intervals, and finally destruction of the elements of perception, the optic fibres and nerve cells. Hence, also, the development of the excuvation of the optic disc by pressing out the substance of the nerve.

3. As it regards simple chronic glaucoma, a type essentially chronic, it is caused by the slow and continuous secretion of this same superabundant liquid, and the very slowness of the effusion will explain perfectly the absence or the feebleness of the pains, the slight obscurations of vision and the lack of trouble of the refracting media, a trouble whose presence, on the contrary, would be easily explained in acute and chronic inflammatory glaucoma.

4. As for *glaucoma fulminans* we must refer it to the sudden and immediate effusion of the greatest possible quantity of liquid, which would at once cause such a forcible compression of the retinal and nervous elements, that they would be immediately paralyzed, so that, the effusion being at length absorbed, as it ordinarily happens, the sight would none the less remain abolished for ever and without remedy.

5. We have also in this the key to a phenomenon, which has been inexplicable up to the present time, the frequency of consecutive glaucoma after a crowd of ocular affections, in which this complication has not been explained heretofore, except by the aid of more or less contestable theories.

6. Finally, and this is the most important point, it appears to me that we have from this standpoint, as we said above, a very plausible explanation of the of therapeutic action of an iridictomy, which we may henceforth compare in every particular with that of a thoracentisis in pleuritic effusions and in general with that of the puncture or evacuation of all effusions in serous cavities.

In fact we know that if iridictomy gives brilliant results in acute glaucoma, and if the cure of this form of the disease is lasting by the means in question, it is not the same in chronic glaucoma, simple or inflammatory. In truth, the cases of this kind in which we are obliged to have recourse to a second iridictomy are numerous, and the therapeutic action of the operation has for a long time been recognized as problematical to such a degree, that its insufficiency has evoked from Coccius the proposition to substitute for simple iridectomy in chronic glaucoma, the imprisoning between the lips of the wound of a portion of the iris, this maneuver having appeared to this author to give much more favorable results.* We must remember, also, how frequent it is to observe a delay in cicatrisation of the sclero-corneal wound or cystoid cicatrisations following an iridectomy in chronic glaucoma.

This gave rise to a very interesting discussion in the Society of Heidelberg in 1869. Von Graefe has called attention anew to these facts in his last and admirable work on glaucoma, which was, if we may be allowed the expression his song of the swan. The readers of the *Annals* having had the good fortune of a complete translation of this *chef d'œuvre* of the master,† we must be contented with referring to it, remarking only that all the remarks which he makes upon the subject which occupies us, accord wonderfully with the actual anatomy.

Let it suffice, in closing, to say that in our view the action of the iridectomy consists only in the evacuation of the effused liquid, and that its action is in all things analagous to that of thoracentesis. Consequently the operation has by so much the greater value as it is done at a time nearer the commencement of the disease, as the sclero-corneal incision is made at a point nearest the extreme limit of the interior chamber, as the section of iris removed is greater, and as this incision is carried to a point adjacent to the ciliary attachment of the iris. And if Coccius has discovered that the enslaving of a portion of the iris in the wound gives more satisfactory results than simple iridectomy in chronic glaucoma, it comes from this, that the portion of the iris enslaved acts the part of a tent, which favors

---

*We ought to remark here that this author has since then renounced this practice, having found that the end was not always attained and that the contrary sometimes happened.

†*Annales d'Oculistique*, vol , LXIII, p. 39, et seq.

the running out of the liquid by capillary attraction or by spónginess.*

A last word still in conclusion. If, as we hope, the preceding lines chance to fix definitely the opinion as regards the nature of glaucoma, we will not claim any merit for it. All the honor belongs to Schwalbe, and it will not be one of the least of the services which have been rendered to practice by the microscope, that it has finally lifted the veil which hid from us this question.

It is none the less true that to our dear and regretted master, Von Gaefe, belongs, we repeat, the honor of having anticipated the microscope. His doctrine concerning glaucoma will contribute powerfully, without any doubt, to render immortal this man of good and great genius.

*These lines were written when I learned that Quaglino had published a number of cases of glaucoma cured by simple peripheral incision without iridectomy.

